# Condition-Specific Molecular Network Analysis Revealed That Flagellar Proteins Are Involved in Electron Transfer Processes of *Shewanella piezotolerans* WP3

**DOI:** 10.1155/2021/9953783

**Published:** 2021-07-29

**Authors:** Dewu Ding, Meineng Wang, Meili Wu, Chengzhi Gan, Pu Wu

**Affiliations:** ^1^School of Mathematics and Computer Science, Yichun University, Yichun 336000, China; ^2^Library of Yichun Vocational Technical College, Yichun 336000, China; ^3^School of Mathematics and Computer Science, Chizhou University, Chizhou 247000, China

## Abstract

Because of the ability to metabolize a large number of electron acceptors such as nitrate, nitrite, fumarate, and metal oxides, *Shewanella* species have attracted much attention in recent years. Generally, the use of these electron acceptors is mainly achieved through electron transfer proteins and their interactions which will dynamically change across different environmental conditions in cells. Therefore, functional analysis of condition-specific molecular networks can reveal biological information on electron transfer processes. By integrating expression data and molecular networks, we constructed condition-specific molecular networks for *Shewanella piezotolerans* WP3. We then identified condition-specific key genes and studied their potential functions with an emphasis on their roles in electron transfer processes. Functional module analysis showed that different flagellar assembly modules appeared under these conditions and suggested that flagellar proteins are important for these conditions. We also identified the electron transfer modules underlying these various environmental conditions. The present results could help with screening electron transfer genes and understanding electron transfer processes under various environmental conditions for the *Shewanella* species.

## 1. Introduction

Cellular biological processes are not maintained by a single molecule but rather the interactions between genes, proteins, metabolites, and many other biological molecules in potential molecular networks [1, 2]. It is generally believed that molecular networks are highly modular, and different network modules can be used to perform different cellular functions. Therefore, identifying and extracting functional modules in a network does not only help to understand the structural and functional relationships of molecular networks but also help to discover the hidden biological rules in the networks. This has many important practical applications, such as inferring function-related or coregulated gene sets [3]. Researchers have proposed many methods to predict functional modules in networks [4].

Since the complex characteristics of organisms are not caused by a single mutation or genetic variation, by changes in the functions of the molecular network, the functional modules within the network will change under different environmental conditions, biological states, and cellular processes [5]. Therefore, it is difficult to obtain condition-specific functional modules through general interaction data alone. Functional modules of molecular networks should be studied by combining condition-specific expression data and molecular networks [6, 7].

**Figure 1:**
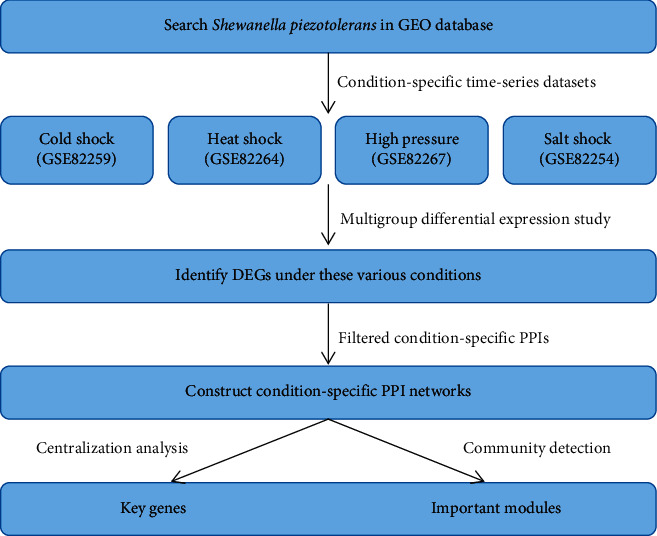
Overall view of the study. Step 1: four time-series expression datasets for *S. piezotolerans* WP3 were obtained from the GEO database; Step 2: the DEGs under these conditions were identified; Step 3: the identified DEGs were used to filter the global PPIs to construct condition-specific PPI networks; Step 4: condition-specific key genes and important modules were identified from these networks. GEO: gene expression omnibus; DEGs: differentially expressed genes; PPI: protein-protein interaction.

Due to their metabolic versatility, *Shewanella* species have attracted much attention, and they have been widely used in many fields, such as energy production, wastewater treatment, bioremediation, biosensor, and chemical synthesis (see, e.g., [8, 9]. It is generally believed that the use of a variety of electron acceptors is mainly achieved through electron transfer proteins (such as multihaem *c*-type cytochromes) and their interactions [10–12]. To reveal the electron transfer mechanism of *Shewanella* species in different environments, the deep-sea bacterium *Shewanella piezotolerans* WP3 (*S. piezotolerans* WP3) was selected in the present work, since it is considered to be a good candidate for studying the adaptation of *Shewanella* species to a variety of environmental conditions (low temperature, high pressure, etc.) [13–15]. Therefore, we integrated expression data and molecular networks of this bacterium under various environmental conditions to construct condition-specific molecular networks and identified the key genes and functional modules in the networks and finally investigated their roles in electron transfer processes (Figure 1). These results will have important significance for screening electron transfer genes and understanding different electron transfer processes in response to various environmental conditions for the *Shewanella* species.

## 2. Materials and Methods

### 2.1. Gene Expression Data

To study electron transfer processes of *S. piezotolerans* WP3 under various conditions, we searched *S. piezotolerans* WP3 in the GEO (gene expression omnibus) database (https://www.ncbi.nlm.nih.gov/geo/) [16] and obtained four time-series expression datasets for the *S. piezotolerans* WP3 wild-type strain under cold shock (GSE82259), heat shock (GSE82264), high pressure (GSE82267), and salt shock (GSE82254) [17, 18]. Each of these datasets contains expression profiling of *S. piezotolerans* WP3 for 30 minutes, 60 minutes, and 90 minutes under the experimental shocks, which could be used for the subsequent differential expression analysis.

### 2.2. Differentially Expressed Genes

In contrast to the comparison of differential expression between two groups of experiments, comparative studies from multigroup data should provide a much better understanding of differential expression [19]. Therefore, comparative studies of multiple groups (three groups of data with 6 replicates for all conditions) were considered in this study. The web tool GEO2R (https://www.ncbi.nlm.nih.gov/geo/geo2r/) [16] was used to compare these multigroup datasets to identify differentially expressed genes (DEGs) under these conditions. The default parameters of GEO2R were used, and *P* < 0.01 and FDR (false discovery rate) <0.05 were used as the cut-off values.

### 2.3. Construction of PPI Networks

Because proteins do not function independently in cells, but generally form complexes with other proteins, that is, protein-protein interaction (PPI) networks, the PPIs stored in STRING (https://string-db.org/) [20] were used for the construction of PPI networks. We first downloaded the full interaction information for *S. piezotolerans* WP3, and to enhance the reliability of the interactions, we extracted the high confidence interactions (combined_score ≥ 0.7, as suggested by STRING) to construct the background PPI network for *S. piezotolerans* WP3. Condition-specific networks can be further obtained by combining this background network with differentially expressed nodes (i.e., the proteins that are encoded by the DEGs).

### 2.4. Network-Based Methods

In general, complex network methods can be used to analyze the structural properties and functions of biological molecule networks. Degree-based centralization analysis was used to study the importance of each protein in the network, with an emphasis on the top 10 key proteins in these networks because proteins generally form complexes with other proteins to perform specific functions, and the ClusterONE method has been shown to be very effective in the recognition of protein complexes [21]. We therefore employed this method to identify the functional modules in these networks. There are several user-friendly implementations of the method (http://www.paccanarolab.org/cluster-one/), and we used the Cytoscape plugin “ClusterONE” to perform the functional module analysis. The minimum size was set to 4, and *P* < 0.05 was used as the cut-off value.

### 2.5. Enrichment Analysis

Enrichment analysis can be commonly used to extract biological information from a given gene/protein set [22]. Here, functional enrichment analysis was performed using the STRING enrichment app through the Cytoscape toolkit (https://cytoscape.org/) [23], and the enrichment FDR cut-off was also set to 0.05. Furthermore, we chose the most representative KEGG pathways from the resulting list (i.e., the lowest FDR), and if there were no enriched KEGG pathways, InterPro enrichment was used instead.

## 3. Results and Discussion

### 3.1. Identification of the DEGs That Are Responsible for the Various Environmental Conditions

We first used GEO2R to identify the DEGs under the various environments, and overall 763, 1635, 1039, and 1130 unique DEGs were involved in cold shock, heat shock, high pressure, and salt shock conditions, respectively. Because proteins need interactions to perform their functions, we constructed the corresponding PPI networks for the proteins encoded by these DEGs. Subsequently, considering that enrichment analysis can be used to extract biological insight from a given gene/protein set, we first performed KEGG pathway enrichment analysis to reveal the biological functions and differences of the largest connected components in these PPI networks.

**Figure 2:**
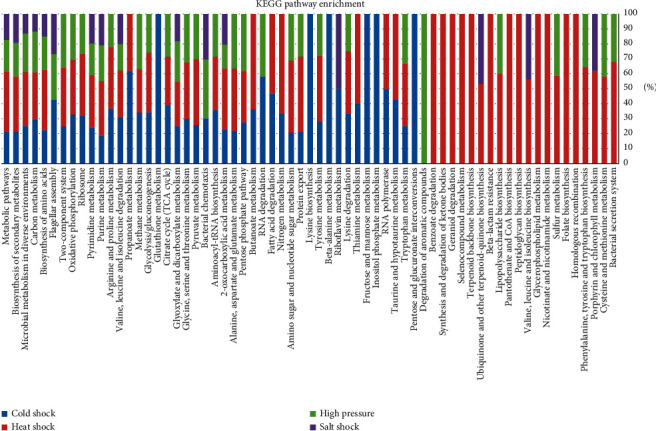
KEGG pathway enrichment analysis for the DEGs (differentially expressed genes) under the various environmental conditions. False discovery rate <0.05.

Overall, 44, 54, 36, and 16 KEGG pathways were enriched under the abovementioned conditions, respectively. As illustrated in Figure 2, the most common enriched pathways across these environments included metabolism-related (e.g., metabolic pathways, biosynthesis of secondary metabolites, and microbial metabolism in diverse environments) and protein translation-related (e.g., biosynthesis of amino acids, ribosomes, aminoacyl-tRNA biosynthesis, and protein export) pathways, indicating that metabolism largely acclimates to changing environmental conditions (cold shock, heat shock, high pressure, and salt shock here), and, accordingly, *S. piezotolerans* WP3 needs to produce new proteins that acclimate to these environments. These results are in good agreement with previous studies in other *Shewanella* species (see, e.g., [24, 25]).

On the other hand, apart from these commonly enriched pathways, some pathways presented large differences across three of these conditions (there were no unique enriched pathways under salt shock condition; see Figure 2). First, the largest difference was derived from the heat shock condition, and there were 12 enriched pathways exclusively presented under this condition, indicating that *S. piezotolerans* WP3 is more sensitive to heat shock than the other shocks examined in this study. This point is in good agreement with the fact that *S. piezotolerans* WP3 was isolated from West Pacific sediment at a depth of ∼1914 m [15], where the most remarkable environmental features are low temperature, high pressure, and high salt, but not high temperature. Second, there were 6 pathways exclusively presented under the cold shock condition, including glutathione metabolism (FDR: 1.35*E* − 05), inositol phosphate metabolism (FDR: 8.54*E* − 03), beta-alanine metabolism (FDR: 1.62*E* − 02), pentose and glucuronate interconversions (FDR: 2.29*E* − 02), lysine biosynthesis (FDR: 2.78*E* − 02), and fructose and mannose metabolism (FDR: 4.58*E* − 02). Third, only one pathway (degradation of aromatic compounds, FDR: 2.86*E* − 02) was exclusively presented under the high-pressure condition. Due to microorganisms need to produce different proteins to cope with the changing environmental conditions, these exclusively enriched pathways and the involved proteins will be helpful to understand the different metabolic processes under various environmental conditions in *S. piezotolerans* WP3.

### 3.2. Construction of the PPI Networks That Are Involved in the Various Environmental Conditions

It is generally believed that the global protein network defines the collective function of cells. In contrast, a condition-specific PPI network contains a lot of interactions that are enriched for processes relevant to the specific condition and thereby may be more relevant for characterizing specific biological processes, such as identifying protein complexes or stimulus-response processes [26, 27].

**Figure 3:**
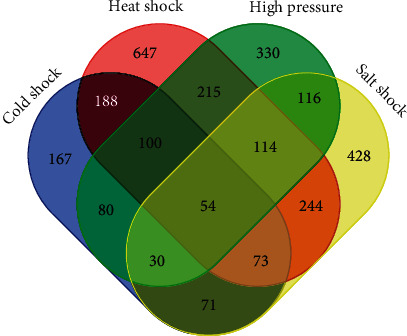
Venn diagram of the differentially expressed genes involved in the various environmental conditions. The figure is produced by using an online Venn diagrams tool (http://bioinformatics.psb.ugent.be/webtools/Venn/).

To construct the PPI networks that are involved in the various environments, we first identified condition-specific protein-coding genes under these conditions by using a Venn diagram. As shown in Figure 3, 167, 647, 330, and 428 unique DEGs were involved in the abovementioned environments, respectively. These gene sets were then used to construct condition-specific PPI networks from the background PPI network for *S. piezotolerans* WP3.

As a result, although there were many isolated proteins in the network, each network contains many interactions that form several interconnected parts. Numerically speaking, all of these condition-specific networks have significantly more interactions than were expected (the PPI enrichment *P* values were 1.66*E* − 2, 7.21*E* − 4, 2.74*E* – 2, and 9.96*E* − 3, respectively). Since proteins mainly carry out biological functions through interactions, it is generally considered that the hubs (i.e., proteins with a high degree value) in the PPI network are more important. To this end, we used the degree value to rank the importance of the proteins in the networks and thereby identified the top 10 key proteins in each network (Table 1). As shown in Table 1, these key proteins are mostly linked to the electron transport processes of *S. piezotolerans* WP3 under various environmental conditions (except for the heat shock condition; see below).

First, the top 10 key proteins under cold shock were mostly relevant to flagellar proteins, including flagellar biosynthesis sigma factor (swp_5124), flagellar basal body rod protein (swp_5105, swp_5110), flagellar basal body L-ring protein (swp_1504, swp_5111), flagellar hook-associated protein FlgL (swp_1508), and flagellar ATPase FliI/YscN (swp_5095). A transcriptional study suggested that the flagellar operon of *Escherichia coli* is inducible in response to cold shock [28]; in addition, a proteomic study showed that flagellar biosynthesis and motility proteins presented significant changes under cold shock [29]. Therefore, the key proteins of *S. piezotolerans* WP3 under cold shock are mostly flagellar proteins and are expectable. Furthermore, the correlation between oxidative electron transport and anaerobic flagellum assembly of *Escherichia coli* was reported long ago [30], and, more importantly, several recent experimental studies from different groups showed that flagellar proteins were involved in the electron transfer activity in *Geobacter sulfurreducens* and *Shewanella oneidensis* (e.g., [31–33], raising a speculation that flagellar proteins may be also involved in the electron transfer activity in *S. piezotolerans* WP3.

Second, the top 10 key proteins under high pressure also included several flagellar proteins, such as flagellar biosynthesis protein (swp_1531) and two flagellar motor switch proteins (swp_1521, swp_1527). Motor switch proteins have been shown to be directly associated with some energy-linked enzymes and thereby lead to higher rates of ATP synthesis, ATP hydrolysis, and electron transport [34]. Other important proteins in this environment are also electron transfer-related, including a flavocytochrome *c* (swp_4352) and a flavoprotein (swp_0430), which are homologous proteins for periplasmic fumarate reductase FccA (63% identity) and quinol:fumarate reductase FAD-binding subunit FrdA (88% identity) from *Shewanella oneidensis* MR-1, respectively. Formate dehydrogenase subunits swp_4312 and swp_2139 are homologous proteins for molybdopterin-binding oxidoreductase (SO_0988, 67% identity) and formate dehydrogenase cytochrome *b* subunit FdhC (47% identity) from *Shewanella oneidensis* MR-1. These proteins play an important role in electron transfer processes of *Shewanella oneidensis* MR-1 [35]. For example, periplasmic fumarate reductase FccA can promote the periplasmic electron transfer process by rapid transient interactions [36].

Third, the most important proteins that are listed in the top 10 key proteins under salt shock are several mannose-sensitive haemagglutinin- (MSHA-) related proteins, including MSHA pilin protein (swp_0495) and MSHA biogenesis proteins (swp_0501, swp_0486), which have been shown to be involved in electron transfer processes in *Shewanella oneidensis* MR-1 [37]. Other notable proteins are *Cbb*_*3*_-type cytochrome oxidase (the oxidase subunit swp_4145 and the cytochrome *c* subunit swp_4144), which are homologous proteins for *Cbb*_*3*_-type cytochrome *c* oxidase subunit CcoN and *Cbb*_*3*_-type cytochrome *c* oxidase subunit CcoO from *Shewanella oneidensis* MR-1. These proteins have previously been shown to be involved in electron transfer in various organisms [38], which made swp_4145 and swp_4144 probable candidates for electron transfer studies in *S. piezotolerans* WP3.

Finally, the top 10 key proteins under heat shock conditions were mainly metabolism-related, including glutamate synthase subunit (swp_3803), pyruvate kinase II (swp_2388), oxaloacetate decarboxylase (swp_1379), multifunctional fatty acid oxidation complex subunit (swp_3139), 2-isopropylmalate synthase (swp_2213), isopropylmalate isomerase small subunit (swp_2216), chorismate mutase (swp_3758), Na^+^-transporting methylmalonyl-CoA/oxaloacetate decarboxylase subunit (swp_1380), sodium pump decarboxylase subunit (swp_1378), and malate dehydrogenase (swp_0481). These results are consistent with previous KEGG enrichment results, indicating that cell metabolism undergoes tremendous changes (from their native living environment) under the heat shock condition. These metabolic proteins may be helpful for identifying which forms of metabolism are more important in response to heat shock.

### 3.3. Important Functional Modules Underlying the Various Environmental Conditions

To identify the functional modules underlying these condition-specific networks and thereby reveal the mechanisms by which *S. piezotolerans* WP3 uses to respond to environmental changes, we used the ClusterONE algorithm to analyze these networks and obtained 6, 13, 10, and 5 functional modules from the abovementioned networks, respectively. We then performed enrichment analysis for these functional modules to reveal their potential functions (see Tables 2–5).

**Figure 4:**
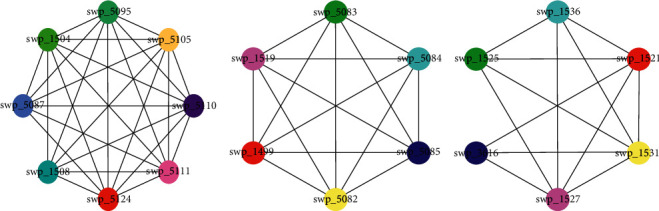
The flagellar assembly module underlying (a) cold shock, (b) heat shock, and (c) high pressure conditions.

First, what interested us was that the different flagellar assembly modules appeared in three of these conditions (Tables 2–5; Figure 4). As we discussed in Section 3.2, flagellar assembly-related proteins are of great concern under cold shock and high-pressure conditions. Although flagellar assembly-related proteins are not key proteins under the heat shock condition, the flagellar assembly module identified here suggests that these proteins should also be important under this condition. As mentioned earlier, flagella are important for biofilm formation, energy production, and electron transfer. The results reported here further illustrate that proteins of flagellar assembly systems will, in fact, form interacting flagellar assembly modules to achieve their purpose. Although they play an important role in a variety of environments, the bacteria can use different flagellar assembly modules under diverse environments, suggesting that diversified flagellar systems are required under diverse environments. In addition, the flagellar assembly module under cold shock contains an important polar flagellum gene (swpt_1508), and the flagellar assembly module under high pressure contains a lateral flagella gene (swp_3616). These results coincide with the fact that *S. piezotolerans* WP3 can express a polar flagellum and multiple lateral flagella systems with distinguishing characteristics [39], which can partly explain how the expression of these systems is differentially regulated under low temperature and high pressure conditions [40].

**Figure 5:**
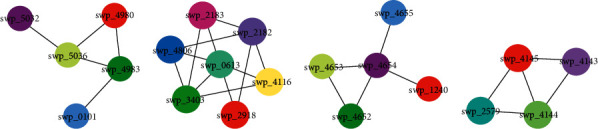
Electron transfer-relevant modules underlying the various environmental conditions. (a) Molybdenum cofactor biosynthesis module under the cold shock condition, (b) multihaem cytochrome module under the heat shock condition, (c) NrfD family module under the high pressure condition, and (d) cytochrome *c* oxidase module under the salt shock condition.

Furthermore, since the use of a variety of electron acceptors is the most important feature of the *Shewanella* species, we then focus on electron transfer processes, and we found several electron transfer-relevant modules in these various environmental conditions (Figure 5).

First, we identified a *molybdenum cofactor biosynthesis module* under the cold shock condition (Figure 5(a)), which could be linked to the fact that some molybdenum cofactor-included enzymes (such as sulfurase) can be used to modulate cold stress-responsive gene expression [41]. Furthermore, the molybdenum cofactor has also been shown to be a redox active prosthetic group, which is an essential component of numerous enzymes for facilitating electron transfer, such as dithiolene complexes [42], aldehyde oxidoreductase [43], and dimethyl sulfide dehydrogenase [44].

Second, a *multihaem cytochrome module* was identified under the heat shock condition (Figure 5(b)). Multihaem cytochromes are major electron transfer proteins in *Shewanella* species. These proteins often contain several closely arranged haem cofactors, which can mediate rapid and long-distance electron transfer by reversible changes between the reduced and oxidized states of iron atoms in haem cofactors [10, 12]. Typically, from the inner membrane through the periplasm and outer membrane to the extracellular space, they can form electron transfer pathways [11]. Recently, we also identified a multihaem cytochrome module in *Shewanella oneidensis* MR-1 under altered O_2_ conditions [25]. Therefore, the multihaem cytochrome module of *S. piezotolerans* WP3 identified here should also be responsible for electron transfer processes under this condition. In addition, the nitrite reductase (cytochrome *c*_*552*_) swp_0613 and swp_3403 can catalyze the reduction of nitrite to ammonia. The reduction of nitrates and nitrites by the shock heating supplies reduced nitrogen under nonreducing conditions, which are necessary for amino acids and nucleic acids, and thus the origin of life under such conditions [45].

Third, an *NrfD family module* was identified under the high-pressure condition (Figure 5(c)), including the formate-dependent nitrite reductase NrfGCD (swp_4654∼swp_4652) and the polysulfide reductase NrfD (swp_1240). Periplasmic nitrite reductase Nrf can obtain electrons from other cytochrome *c* molecules located in the inner membrane and then use them to reduce soluble substances such as nitrites, a process that has been widely studied in *Shewanella oneidensis* MR-1 [46]. Polysulfide reductase NrfD could also be used to transfer electrons from the quinone pool.

Finally, we identified a *cytochrome c oxidase module* under the salt shock condition (Figure 5(d)). The module mainly contains two *cbb*_*3*_-type cytochrome *c* oxidase subunits (swp_4145∼swp_4144) and one class I cytochrome *c* subunit (swp_4143), which are homologous to CcoN, CcoO, and CcoP from *Shewanella oneidensis* MR-1, respectively. Considering that *cco* family of proteins plays a key role in both aerobic and anaerobic respiration in *Shewanella oneidensis* MR-1, and they have also been shown to be involved in the generation of electric current [38], the cytochrome *c* oxidase module is thereby also considered to be related to these processes in *S. piezotolerans* WP3.

## 4. Conclusion

Currently, constructing a network model and then using module detection methods to group genes/proteins is one of the most common methods for genome-scale expression data study [47]. However, networks generally constantly change under different environmental conditions, such as different temperatures and pressures. The study of condition-specific and even dynamic molecular networks (and their functional modules) has become an important topic in bioinformatics researches [6, 7].

To explore the electron transfer mechanism of *Shewanella* species under various environments, we integrated multiexpression data and constructed condition-specific molecular networks for *S. piezotolerans* WP3. We then identified the key electron transfer genes and analyzed the functional modules, both of which suggest that flagellar proteins are important under these conditions. Finally, we discussed the condition-specific electron transfer-relevant modules under these various environmental conditions. Further experimental investigation, such as the influence of the respiratory capacity of the cells through flagellar gene deletion mutant, is needed to verify the putative roles of flagellar proteins in *S. piezotolerans* WP3.

## Figures and Tables

**Table 1:** The top 10 key proteins in the condition-specific PPI networks of *S. piezotolerans* WP3 under various environmental conditions.

Rank	Cold shock	Heat shock	High pressure	Salt shock
1	swp_5124	swp_3803	swp_2623	swp_0485
2	swp_5105	swp_2388	swp_4312	swp_0361
3	swp_1504	swp_1379	swp_5025	swp_2579
4	swp_5095	swp_3139	swp_4352	swp_4934
5	swp_5110	swp_2213	swp_0430	swp_2897
6	swp_5111	swp_2216	swp_2139	swp_0501
7	swp_1508	swp_3758	swp_0854	swp_0495
8	swp_5087	swp_1380	swp_4933	swp_0486
9	swp_2950	swp_1378	swp_1531	swp_4145
10	swp_0586	swp_0481	swp_1521, swp_1527	swp_4144

Note: as many proteins have the same degree value, we used the clustering coefficient as the second measurement to eliminate the proteins that are not ranked in the top 10.

**Table 2:** The most representative KEGG pathway that was enriched in the modules identified from the cold shock condition.

Cluster	Size	Density	*P* value	KEGG pathways	Members
1	8	0.96	1.87*E* − 04	Flagellar assembly	swp_5105, swp_5124, swp_5111, swp_5110, swp_1504, swp_5095, swp_5087, swp_1508
2	7	0.52	8.66*E* − 04	Ribosome	swp_1665, swp_2026, swp_1999, swp_1429, swp_3015, swp_2462, swp_1221
3	7	0.52	1.90*E* − 03	Oxidative phosphorylation	swp_0794, swp_4826, swp_4824, swp_2950, swp_2059, swp_2948, swp_5156
4	5	0.50	3.54*E* − 03	**Molybdenum cofactor biosynthesis**	swp_5032, swp_5036, swp_4980, swp_4983, swp_0101
5	5	0.50	7.98*E* − 03	Propanoate metabolism	swp_3440, swp_4179, swp_3386, swp_0423, swp_1949
6	5	0.60	2.94*E* − 02	Aminoacyl-tRNA biosynthesis	swp_3015, swp_2462, swp_3260, swp_3938, swp_0586

Note: the bold enrichment term indicates that there was no KEGG enrichment and InterPro enrichment was used alternatively.

**Table 3:** The most representative KEGG pathway that was enriched in the modules identified from the heat shock condition.

Cluster	Size	Density	*P* value	KEGG pathways	Members
1	15	0.67	2.85*E* − 05	Pyruvate metabolism	swp_1380, swp_5027, swp_4752, swp_4333, swp_3528, swp_2388, swp_2216, swp_2215, swp_2213, swp_2053, swp_1378, swp_1379, swp_4077, swp_0481, swp_0360
2	12	0.56	1.56*E* − 04	Ribosome	swp_2008, swp_2030, swp_4802, swp_3049, swp_2027, swp_2028, swp_2025, swp_2007, swp_0208, swp_1998, swp_0427, swp_1556
3	9	0.64	2.84*E* − 04	Peptidoglycan biosynthesis	swp_2592, swp_2234, swp_3922, swp_2293, swp_2225, swp_4399, swp_2229, swp_2228, swp_2224
4	6	1.00	6.31*E* − 04	Flagellar assembly	swp_1499, swp_5085, swp_5084, swp_5083, swp_5082, swp_1519
5	7	0.62	7.64*E* − 04	Beta-lactam resistance	swp_1046, swp_4921, swp_4710, swp_2733, swp_1301, swp_1300, swp_2423
6	6	0.73	1.28*E* − 03	**Na^+^-translocating NADH-quinone reductase**	swp_1172, swp_2888, swp_2886, swp_2884, swp_1173, swp_2883
7	7	0.62	1.94*E* − 03	**Multihaem cytochrome**	swp_4806, swp_2182, swp_4116, swp_2918, swp_2183, swp_3403, swp_0613
8	8	0.64	2.70*E* − 03	Lipopolysaccharide biosynthesis	swp_3507, swp_3506, swp_0834, swp_3511, swp_3509, swp_3508, swp_3516, swp_3515
9	5	0.70	2.79*E* − 03	—	swp_3204, swp_3981, swp_0083, swp_3979, swp_1828
10	4	0.67	1.01*E* − 02	—	swp_2103, swp_3188, swp_3190, swp_3192
11	5	0.70	1.71*E* − 02	Glycerophospholipid metabolism	swp_0081, swp_5056, swp_4820, swp_4318, swp_0775
12	5	0.60	2.91*E* − 02	Bacterial secretion system	swp_0196, swp_0739, swp_0187, swp_0189, swp_0188
13	4	0.67	3.34*E* − 02	—	swp_3543, swp_4396, swp_0518, swp_0110

Note: the bold enrichment terms indicate that there was no KEGG enrichment and InterPro enrichment was used alternatively; — indicate that the enrichment analysis returned with no results.

**Table 4:** The most representative KEGG pathway that was enriched in the modules identified from the high-pressure condition.

Cluster	Size	Density	*P* value	KEGG pathways	Members
1	9	0.58	3.49*E* − 04	Carbon metabolism	swp_3663, swp_5025, swp_4312, swp_1239, swp_2139, swp_2142, swp_3875, swp_3458, swp_0182
2	9	0.50	9.74*E* − 04	Oxidative phosphorylation	swp_4058, swp_0854, swp_4352, swp_3589, swp_4940, swp_1424, swp_1425, swp_0430, swp_0429
3	6	0.87	1.26*E* − 03	Flagellar assembly	swp_1531, swp_3616, swp_1536, swp_1521, swp_1527, swp_1525
4	4	0.67	1.01*E* − 02	—	swp_0679, swp_4217, swp_0678, swp_2705
5	4	0.67	1.01*E* − 02	—	swp_0059, swp_0060, swp_0056, swp_1586
6	5	0.50	1.42*E* − 02	—	swp_4653, swp_4654, swp_4652, swp_4657, swp_4655
7	7	0.52	2.19*E* − 02	—	swp_2623, swp_2495, swp_4933, swp_4301, swp_4936, swp_4935, swp_4940
8	4	0.50	2.89*E* − 02	Purine metabolism	swp_1361, swp_4724, swp_2972, swp_2621
9	5	0.50	4.13*E* − 02	**NrfD family**	swp_4653, swp_4654, swp_4652, swp_4655, swp_1240
10	6	0.53	4.29*E* − 02	Microbial metabolism in diverse environments	swp_1907, swp_2623, swp_1982, swp_4429, swp_5068, swp_1640

Note: the bold enrichment term indicates that there was no KEGG enrichment, and InterPro enrichment was used alternatively; — indicate that the enrichment analysis returned no results.

**Table 5:** The most representative KEGG pathway that was enriched in the modules identified from the salt shock condition.

Cluster	Size	Density	*P* value	KEGG pathways	Members
1	4	0.67	1.01*E* − 02	**Protein of unknown function DUF3131**	swp_2164, swp_2170, swp_2165, swp_2169
2	4	0.83	2.35*E* − 02	**Cytochrome c oxidase subunit**	swp_2579, swp_4145, swp_4144, swp_4143
3	4	1.00	3.01*E* − 02	—	swp_0495, swp_0501, swp_0486, swp_0485
4	4	0.67	3.34*E* − 02	—	swp_2897, swp_2732, swp_3465, swp_2421
5	4	0.67	4.75*E* − 02	Valine, leucine and isoleucine biosynthesis	swp_2214, swp_2842, swp_0361, swp_2606

Note: the bold enrichment terms indicate that there was no KEGG enrichment and InterPro enrichment was used alternatively; — indicate that the enrichment analysis returned no results.

## Data Availability

All relevant data are within the paper.
